# A Review of Solvate Ionic Liquids: Physical Parameters and Synthetic Applications

**DOI:** 10.3389/fchem.2019.00263

**Published:** 2019-04-18

**Authors:** Daniel J. Eyckens, Luke C. Henderson

**Affiliations:** Institute for Frontier Materials, Deakin University, Geelong, VIC, Australia

**Keywords:** solvate ionic liquid, lithium TFSI, glyme complexes, chelation, solvatochromic properties

## Abstract

Solvate Ionic Liquids (SILs) are a relatively new class of ionic liquids consisting of a coordinating solvent and salt, that give rise to a chelate complex with very similar properties to ionic liquids. Herein is the exploration of the reported Kamlet-Taft parameters, Gutmann Acceptor numbers and the investigation of chelating effects through NMR spectroscopy of multiple atomic nuclei. These properties are related to the application of SILs as reaction media for organic reactions. This area is also reviewed here, including the implication in catalysis for the Aldol and Kabachnik-Fields reactions and electrocyclization reactions such as Diels-Alder and [2+2] cycloaddition. Solvate ILs exhibit many interesting properties and hold great potential as a solvent for organic transformations.

## Introduction/Context

The context of this review is to examine the application of solvate ionic liquids as a reaction media. This extends to encompass the relevant physical parameters of hydrogen bonding characteristics, polarity and Lewis acidity. The use of these solvates as electrolytes for lithium ion batteries (Ueno et al., 2013; Kido et al., 2015; Nakazawa et al., 2016; Kawazoe et al., 2017), their microstructure (Murphy et al., 2016; Saito et al., 2016; Cook et al., 2017; Li H. et al., 2017) or indeed their thermoelectrochemical properties (Black et al., 2018) are not covered here. In addition to these applications, it may be of interest to note that they have also been evaluated as sizing agents for carbon fiber in resin composites, with good success (Eyckens et al., 2018).

It is important to note that while this review focusses on the use of SILs in organic and materials chemistry there has been substantial use of traditional ILs in both of these fields. There has been considerable work in the use of ionic liquids in the modification of graphene with imidazolium ionic liquids, one reporting a one-step functionalization procedure from graphite (Liu et al., 2008). The use of these materials has also seen a great amount of application in sensors of biological species such as NADH (Shan et al., 2010; Atta et al., 2017), hydrogen peroxide (Chen et al., 2018), bisphenol A (Wang et al., 2018), and glucose (Zhang et al., 2011). These materials have also seen use in nanocomposites still as electrodes for detection of chemical or biological species (Krampa et al., 2017; Li J. et al., 2017; Zad et al., 2018).

The work in materials science is also not limited to imidazolium-derived ionic liquids and interesting works in graphene exfoliation have been conducted in pyridinium and pyrrolidinium cation based ionic liquids (Chaban and Fileti, 2015). Examination of the difference between molecular liquids and ionic liquids has been reported (Bordes et al., 2018) and followed by exploring the difference between *N*-butylpyridinium *bis*(trifluoromethanesulfonyl)imide and 1-butyl-1-methylpyrrolidinium *bis*(trifluoromethanesulfonyl)imide (Chaban et al., 2017). Both of these ionic liquids were superior to the imidazolium-derived ionic liquid 1-ethyl-3-methylimidazolium tetrafluoroborate by ~20 kJ mol^−1^ nm^−1^. Complementing this work is other investigations into pyridinium and pyrrolidinium-derived ionic liquids in various fields such as heat transfer processes (Musiał et al., 2017), super capacitors (Chee et al., 2016; Chaban et al., 2018), and graphene nanostructures (Atilhan and Aparicio, 2014).

### Solvate Ionic Liquids

**Figure 1:**
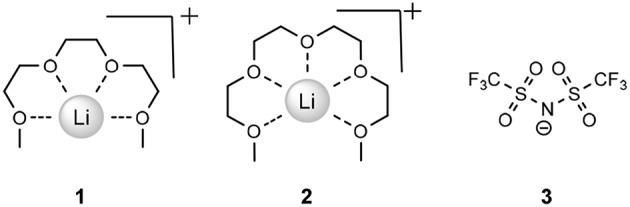
Simplified structure of Lithium in G3 ([Li(G3)]TFSI, **1**), G4 ([Li(G4)]TFSI, **2**), and the TFSI^−^ anion (**3**).

A recently reported (Tamura et al., 2010) class of ionic liquids known as solvate ionic liquids (SILs) is an area of study in its infancy, though investigations into to the field are rapidly increasing. Typically, SILs exist due to the diffusing of a cationic charge *via* the sequestering of a hard cation, often in an ethereal solvent (Austen Angell et al., 2012). The most prominent in the literature is the dissolution of lithium *bis*(trifluoromethanesulfonyl)imide (LiTFSI) in either triethylene glycol dimethyl ether or tetraethylene glycol dimethyl ether ([Li(G3)]TFSI (**1**) or [Li(G4)]TFSI (**2**), respectively, Figure 1).

The use of ionic liquids for organic transformations (Welton, 1999; Hallett and Welton, 2011), and their physical properties (Forsyth et al., 2002; Angell et al., 2007; Lee et al., 2008; Ferrara et al., 2017) have been well studied, though these solvate counterparts have a relatively low representation in the literature. Also well-established is the effect on reaction kinetics the use of ILs convey (Keaveney et al., 2017; Butler and Harper, 2018; Hawker et al., 2018), an area that has had, at the time of publishing, not been investigated in SILs.

The LiTFSI salt has been incorporated into polymer electrolytes for batteries, with good success (Watanabe and Nishimoto, 1995; Mary Sukeshini et al., 1996; Watanabe and Mizumura, 1996; Nishimoto et al., 1998), and the addition of boric acid ester monomers have assisted the solubility of the salt in these networks (Hirakimoto et al., 2001; Tabata et al., 2003). Henderson et al. first identified and characterized the chelation of Li^+^ salts in triglyme and tetraglyme (Henderson et al., 2003) but the salts studied in that work didn't include LiTFSI.

Both [Li(G3)]TFSI and [Li(G4)]TFSI satisfy the following criteria for solvate ILs outlined by Mandai et al. (2014): (1) A solvate compound is formed between an ion and a ligand(s) in a certain stoichiometric ratio (in this instance, 1:1). (2) Consist (almost) entirely of complex ions (solvates) and their counter ions in the molten state. (3) Show no physicochemical properties based on both pure ligands and precursor salts under using conditions. (4) Have a melting point below 100°C (which satisfies the criterion for typical room temperature ILs). (5) Have a negligible vapor pressure.

In relation to the third criterion, the equimolar mixture of LiTFSI and glyme forgoes any evaporation due to the lithium chelation, an effect not present when lesser concentrations of LiTFSI in glyme were assessed (Mandai et al., 2014).

**Scheme 1:**
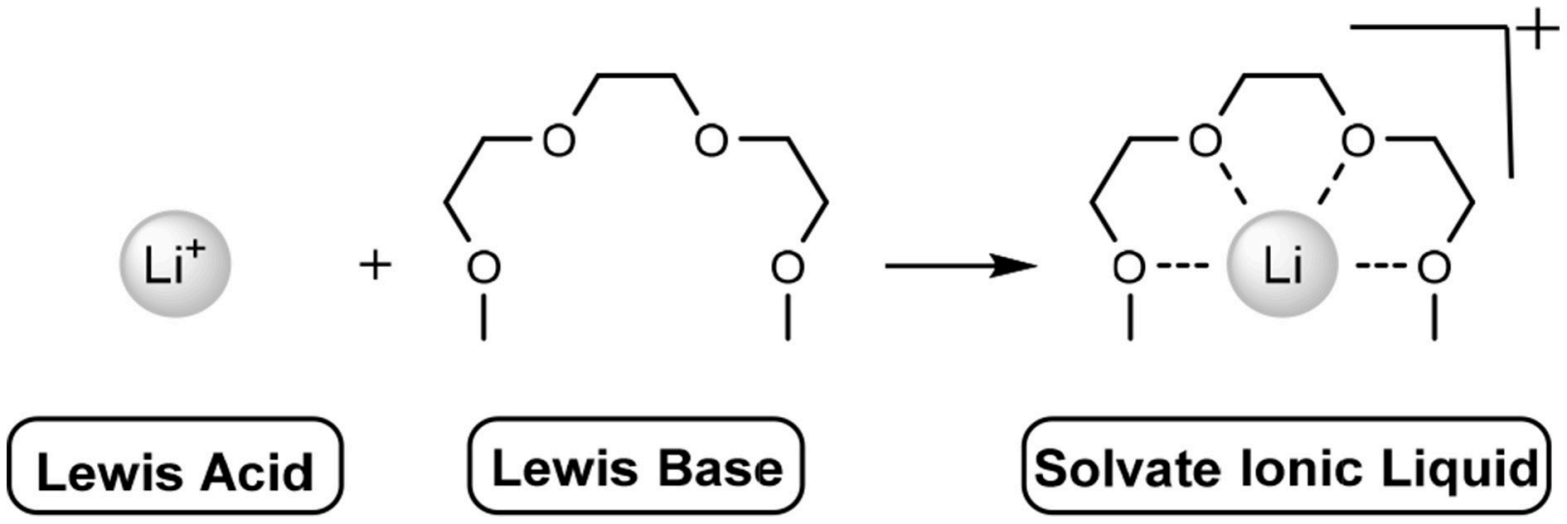
Addition of Lewis acid and Lewis base to give a solvate ionic liquid. Anion not shown.

The solvation occurs when the lone pairs on the oxygen atoms of the ether moieties act as a Lewis base, donating electrons to the Lewis acid (lithium) cation, chelating it (Scheme 1). This multidentate sequestration enables the dissolution of alkali metals, such as LiTFSI or lithium perchlorate (LiClO_4_). In the case of LiTFSI, electron density of the nitrogen atom (of the anion) is attenuated due to the strongly electron withdrawing flanking groups [sulfur(VI)], heavily delocalising the anionic charge. This allows the oligo-ether groups to coordinate the lithium cation. The combination of these effects relieves the strong coulombic interactions of the naked LiTFSI salt, reducing the previously high melting point (234–238°C) to below room-temperature.

**Scheme 2:**
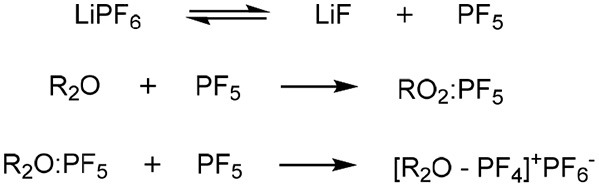
Mechanism of decomposition of Glyme-like groups (R_2_O) through reaction with the PF_6_ anion (Abraham et al., 1997).

Changing the glyme or anion combination can produce highly variable results, exemplified by the crystal structure produced when LiClO_4_ is dissolved in G3 (melting point = 103°C) compared with the room-temperature IL formed when dissolved in G4 (Ueno et al., 2012). The greater the ionic association of the salt in aprotic solvents, the less likely that chelation will occur, supported by the previous example by the greater Lewis basicity (association with cation) of the anion ${\text{ClO}}_{4}^{-}$ compared to TFSI^−^ (Henderson, 2006). Further to this, employing a ${\text{PF}}_{6}^{-}$ anion may catalyze the decomposition of polyethylene oligomers to alkenes, with concimmitant HF and phosphorous containing by-products, according to Scheme 2 (Abraham et al., 1997).

### Background

**Scheme 3:**
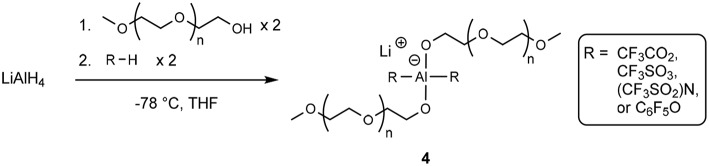
Production of an aluminate-based lithium solvate salt (Fujinami and Buzoujima, 2003).

Similar structures of Li^+^ cations with oligoether-containing structures were investigated by Fujinami and Buzoujima (2003) which consisted of two ethereal units and two electron withdrawing units attached to an aluminate anionic center (**4**) according to Scheme 3.

**Figure 2:**
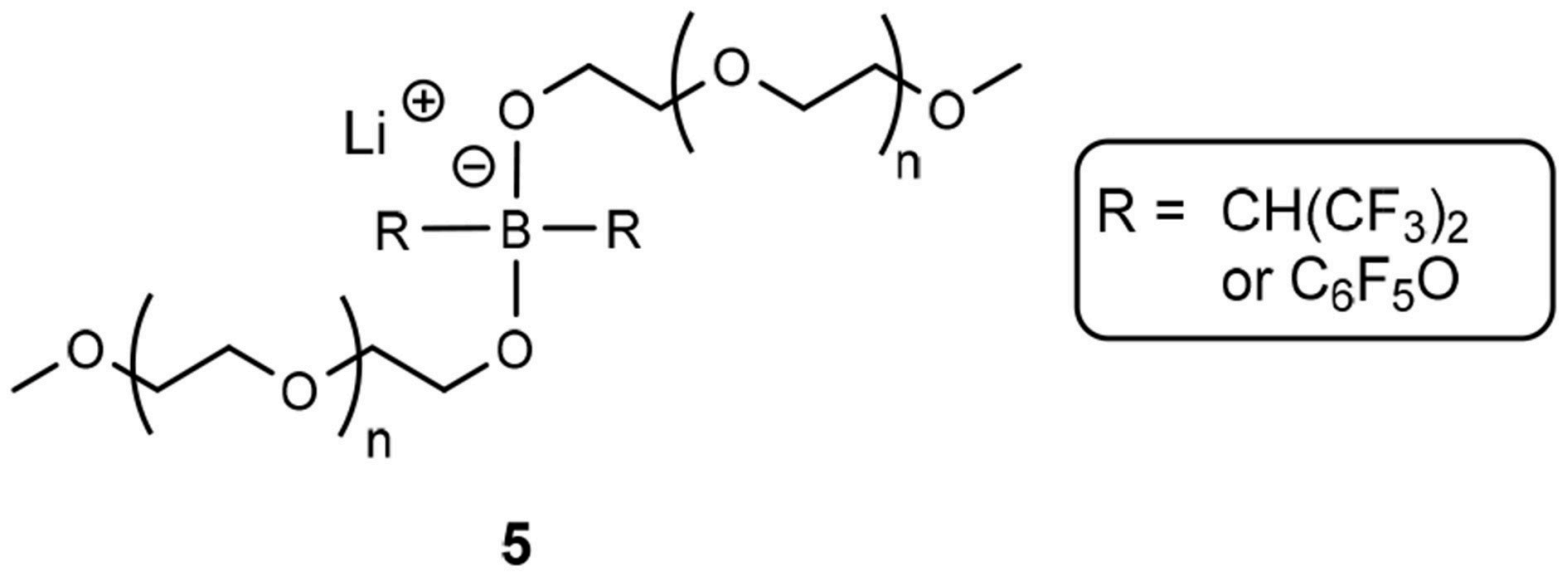
Borate-based solvate ionic liquid produced by Shobukawa et al. (2004).

Comparatively, Shobukawa et al. demonstrated an analogous structure, though with a borate anionic center (**5**, Figure 2) (Shobukawa et al., 2004). The main difference here is that the latter example was fully characterized as an ionic liquid, and the former (aluminate solvate), was not formally characterized, though was referred to as a liquid salt in the manuscript (Fujinami and Buzoujima, 2003). Both structures exhibit similar glass transition states, far below 0°C (average T_g_ ≈ −50°C across different length PEG groups) and the reduction in Lewis basicity of the anionic component of these solvates allow the Li^+^ cation to be chelated by the ethereal units (Shobukawa et al., 2004).

Although these examples are similar in concept to the solvate ionic liquids explored here, Pappenfus et al. was the first to report the equimolar mixture of LiTFSI and tetraglyme as a room temperature (solvate) ionic liquid (Pappenfus et al., 2004). This was closely followed by the analysis of different length glyme oligomers with lithium salts (Henderson, 2006).

The chelation of the lithium cation by the glyme molecule characterizes the mixture as a solvate ionic liquid, not just a concentrated solution, as identified by Ueno et al. (2012) by exploring anion-dependent properties. It was determined that a weakly Lewis basic anion (TFSI-type anions and ClO_4_) was required to allow the glyme-Li^+^ chelation to dominate over the competitive cation-anion interactions. When anions of greater Lewis basicity were employed, the result was concentrated solutions of salt in glyme (Table 1, entries with asterisks).

**Table 1:** Properties of a range glyme-lithium salt mixtures.

**Glyme-Li^**+**^ salt mixture**	**T_**m**_ (^**°**^C)**	**ρ (g cm^**−3**^)**
**[Li(G3)]TFSI**	**23**	**1.42**
[Li(G3)]FSI	56	1.36
[Li(G3)]OTf^*^	35	1.30
[Li(G3)]${\text{NO}}_{3}^{*}$	27	1.18
[Li(G3)]TFA^*^	n.d.	1.20
**[Li(G4)]TFSI**	**n.d**.	**1.40**
[Li(G4)]CTFSI	28	1.40
[Li(G4)]FSI	23	1.32
[Li(G4)]BETI	23	1.46
[Li(G4)]ClO_4_	28	1.27
[Li(G4)]BF_4_	39	1.22
[Li(G4)]${\text{NO}}_{3}^{*}$	n.d.	1.17
[Li(G4)]TFA^*^	n.d.	1.19

*Entries in bold are those most relevant to this review*.

* *denotes concentrated solutions, not solvate ILs. TFSI, bis(trifluormethansulfonyl)imide; FSI, bis(fluorosulfonyl)imide; OTf, trifluoromethylsulfonate; NO_3_, nitrate; TFA, trifluoroacetate; CTFSI, cyclic-TFSI derivative 1,2,3-dithiazolidine-4,4,5,5-tetrafluoro-1,1,3,3-tetraoxide; BETI, bis(pentafluoroethanesulfonyl)imide; ClO_4_, perchlorate; BF_4_, tetrafluoroborate; Adapted from Ueno et al. (2012)*.

In contrast to the concentrated solutions, the solvate ILs showed high thermal stability, high ionic conductivity, high viscosity, low volatility, low flammability as well as a wide electrochemical window, just like traditional ILs (Tamura et al., 2010; Seki et al., 2011; Ueno et al., 2012).

Table 1, below, shows the difference in melting point and density of equimolar mixtures of tri- or tetraglyme and lithium salts with a variety of anions.

As may be seen in the table, altering the anion has large effects on both melting point and density. The solvate ionic liquids explored in this work are in bold, and worth noting is the outcome that not all lithium salts present as liquids in both tri- and tetraglyme, and may only exist as such in one or the other with the alternative being a solid complex. These combinations are not shown in Table 1, but an example is [Li(G3)]ClO_4_ which is solid above 100°C, whereas [Li(G4)]ClO_4_ has a melting point of 28°C.

**Figure 3:**
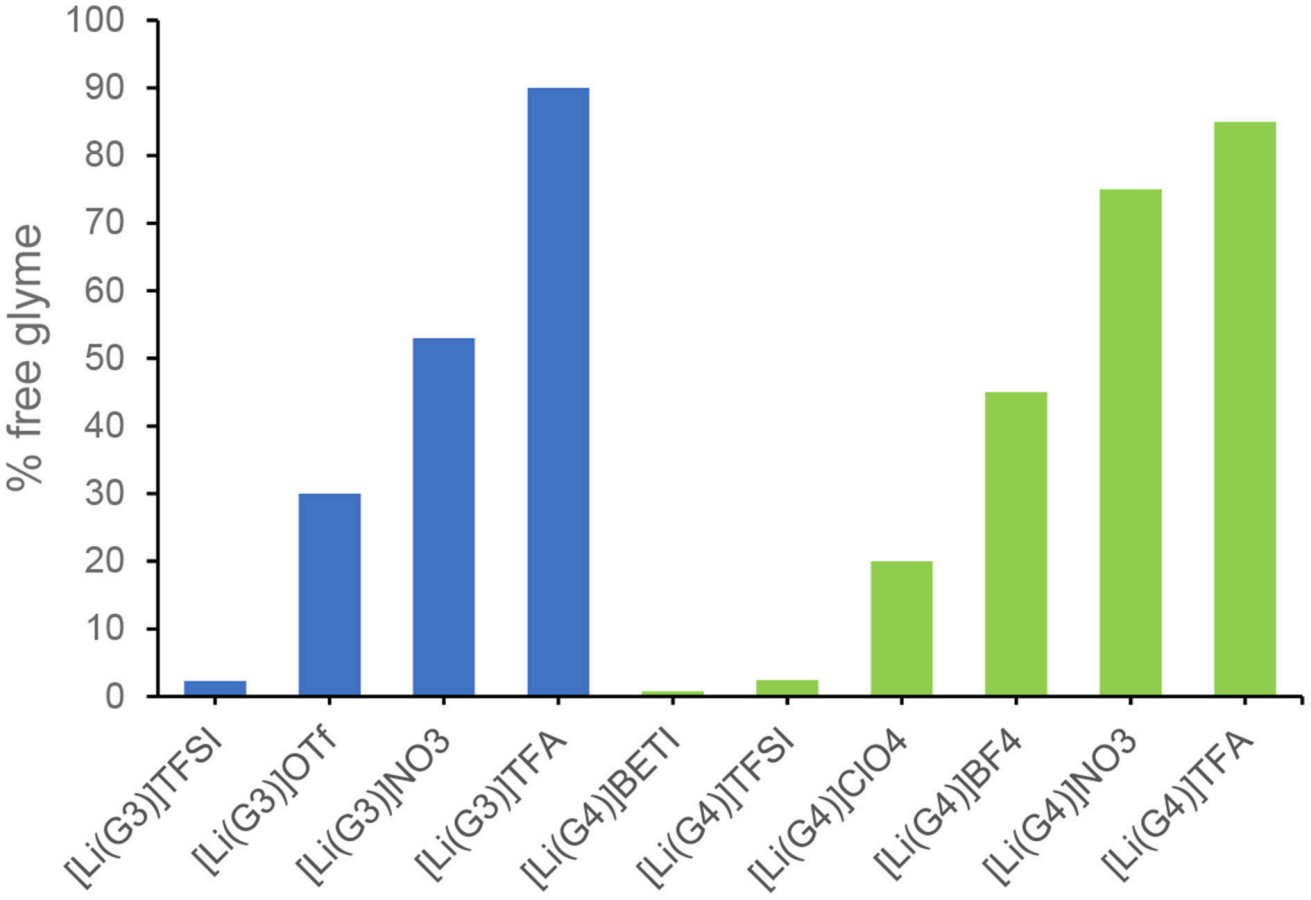
Estimated amount of free glyme in solution for equimolar mixtures of tri- or tetraglyme with various lithium salts.

Raman spectroscopy of both [Li(G3)]TFSI and [Li(G4)]TFSI indicates a very negligible percentage of free glyme in these equimolar mixtures (Figure 3), which is to be expected of solvate ILs (Ueno et al., 2015).

As identified earlier, some combinations of lithium salt and glyme do not result in solvate ionic liquids, only concentrated solutions, further confirmed by the large percentage of free glyme (not-chelating) in those examples (Ueno et al., 2015).

## Physical Chemical Properties

### Kamlet-Taft Parameters

Sometimes referred to as solvatochromic parameters, the Kamlet-Taft parameters are determined spectrophotometrically *via* the addition of different dyes to solvents and subsequent UV-visible spectroscopy (Kamlet et al., 1977). The scale is based on linear solvation energy relationships (LSER) consisting of three complimentary solvent characteristics. These are the hydrogen-bond donating and accepting abilities (α and β values, respectively) and polarity/polarizability (π^*^ values). It is also possible to determine the E_T_(30) (solvent polarity) and the $E_{T}^{N}$ (solvent polarity normalized against water) from one of the dyes used (Reichardt's dye).

This technique has previously been employed in molecular solvents (Kamlet and Taft, 1976; Taft and Kamlet, 1976; Kamlet et al., 1977), in non-aqueous binary mixtures of organic solvents (Reta et al., 2001), as well as for ionic liquids (Muldoon et al., 2001; Lee et al., 2008; Padró and Reta, 2016). In addition to the key characteristics that can be investigated through this technique, is the reactivity of anionic nucleophiles in ionic liquids (Crowhurst et al., 2006). All solvents must be appropriately anhydrous when employing these techniques, to reduce any augmentation of results.

The determination of the physical parameters of SILs is still in its infancy, though in regard to the Kamlet-Taft parameters, there have been three reports in the literature (Dolan et al., 2016; Eyckens et al., 2016b; Black et al., 2018). Each have had some slight difference in detection method, be it different dyes or different mole ratio of solvating glyme to LiTFSI salt. Due to this fact, the reported values have some slight variation from one another, though it is important to note that the trend of these parameters is of most importance when comparing these values, which is largely consistent.

**Figure 4:**
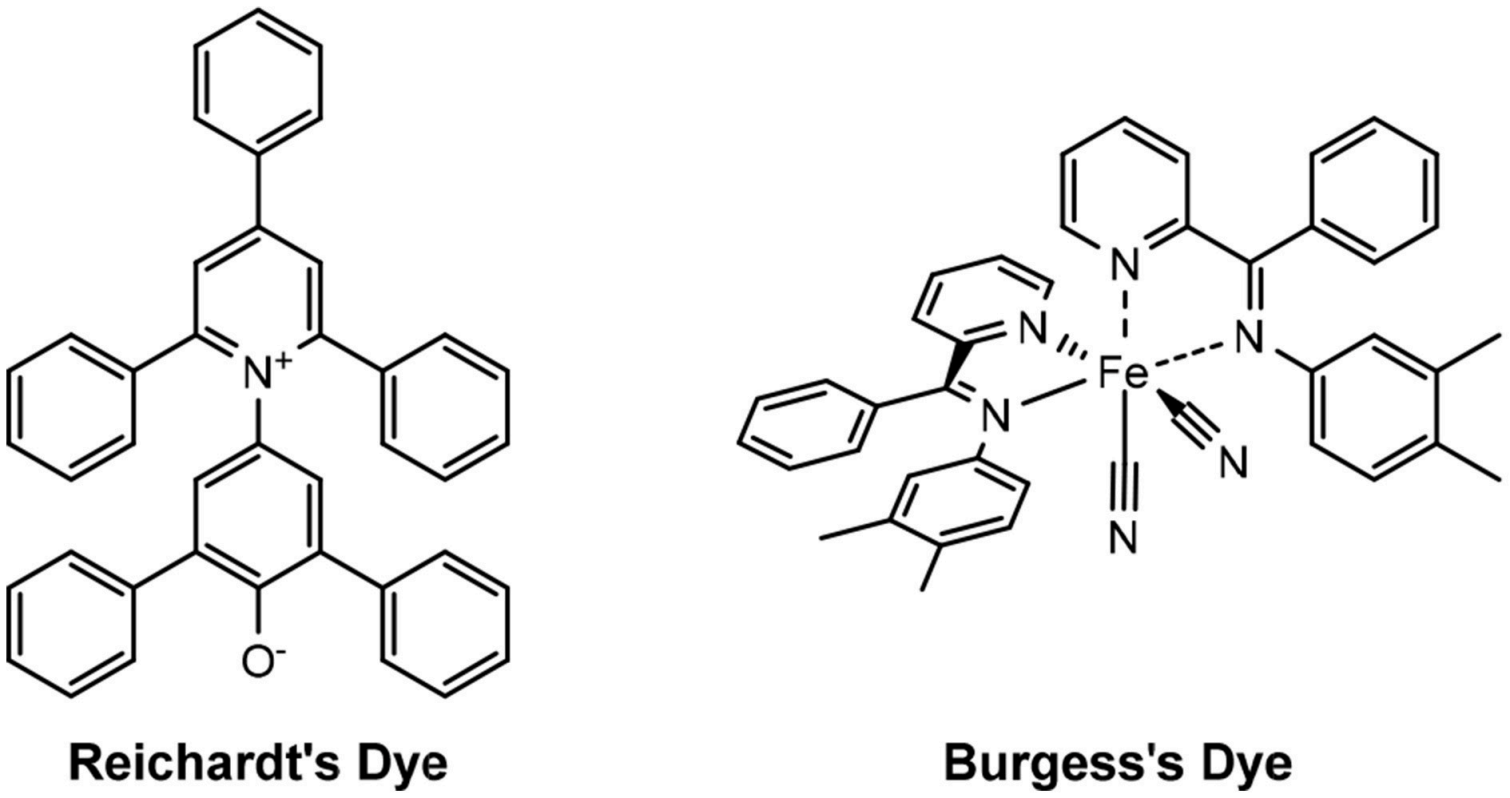
Structures of Reichardt's and Burgess's dyes.

The dyes used to determine the Kamlet-Taft parameters are traditionally *N,N*-diethyl-4-nitroaniline, 4-nitroaniline and Reichardt's dye. Burgess' dye may be used in place Reichardt's dye (Figure 4), if the latter is not sufficiently soluble in the given solvent, as was the case for Dolan et al. except for the glyme solvents (Table 2) (Dolan et al., 2016).

**Table 2:** Reported Kamlet-Taft parameters of solvate ionic liquids.

**Solvent**	**α**			**β**			π^*^		
	**(i)**	**(ii)**	**(iii)**	**(i)**	**(ii)**	**(iii)**	**(i)**	**(ii)**	**(iii)**
G3	0.10	0.04	−0.01	0.62	0.66	0.71	0.74	0.66	0.67
G4	0.03	0.02	−0.05	0.72	0.66	0.72	0.68	0.66	0.69
[Li(G3)]TFSI	1.03^a^	1.08	1.32	0.30	0.26	0.31	0.96	1.00	0.94
[Li(G4)]TFSI	0.77^a^	1.22^b^	1.35	0.24	0.27^b^	0.28	0.92	0.99^b^	0.91
[Li(G3)]BETI	0.88^a^	-	-	0.32	-	-	0.84	-	-
[Li(G4)]BETI	0.80^a^	-	-	0.36	-	-	0.86	-	-
[Bmim]TFSI	-	-	0.55	-	-	0.22	-	-	0.97

*(i) (Dolan et al., 2016) (ii) (Black et al., 2018) (iii) (Eyckens et al., 2016b)*.

a *Values determined with Burgess' dye in place of Reichardt's dye*.

b *Mole ratio of 4:5 (LiTFSI:G4) used instead of 1:1*.

The solvents examined here are the neat glymes (tri- and tetraglyme, G3 and G4, respectively), LiTFSI versions of these ([Li(G3)]TFSI and [Li(G4)]TFSI), the LiBETI (lithium *bis*(pentafluoroethanesulfonyl)imide) versions ([Li(G3)]BETI and [Li(G4)]BETI), and for comparison to a traditional ionic liquid, [Bmim]TFSI (1-butyl-3-methylimidazolium *bis*(trifluoromethanesulfonyl)imide). For greater investigation of anion combinations, please refer to the literature (Dolan et al., 2016). Solvates consisting of the LiBETI salt are presented as the G4 varietal is reported to have a very low percentage of free glyme (Table 1) (Ueno et al., 2015), and though this hasn't been reported for [Li(G3)]BETI (potentially due to the higher melting point, mp = 74°C) it has been included here for consistency.

The Kamlet-Taft parameters are measured on a scale generated by examining the wavelength exhibited by solvents of differing hydrogen bond characteristics in response to the presence of different dyes. Due to this, the values are measured in reference to a strongly hydrogen bond donating or accepting solvent, which is given the arbitrary value of 1.00. It is possible that a solvent of unknown hydrogen bonding properties may have a value of <1.00, meaning for example that it is a greater hydrogen bond donator than the given reference.

#### α-Values

Looking first at the α values (hydrogen bond donating), the tri- and tetraglyme both exhibit negligible values across all reports, as is anticipated judging from their structures and lack of acidic protons.

**Figure 5:**
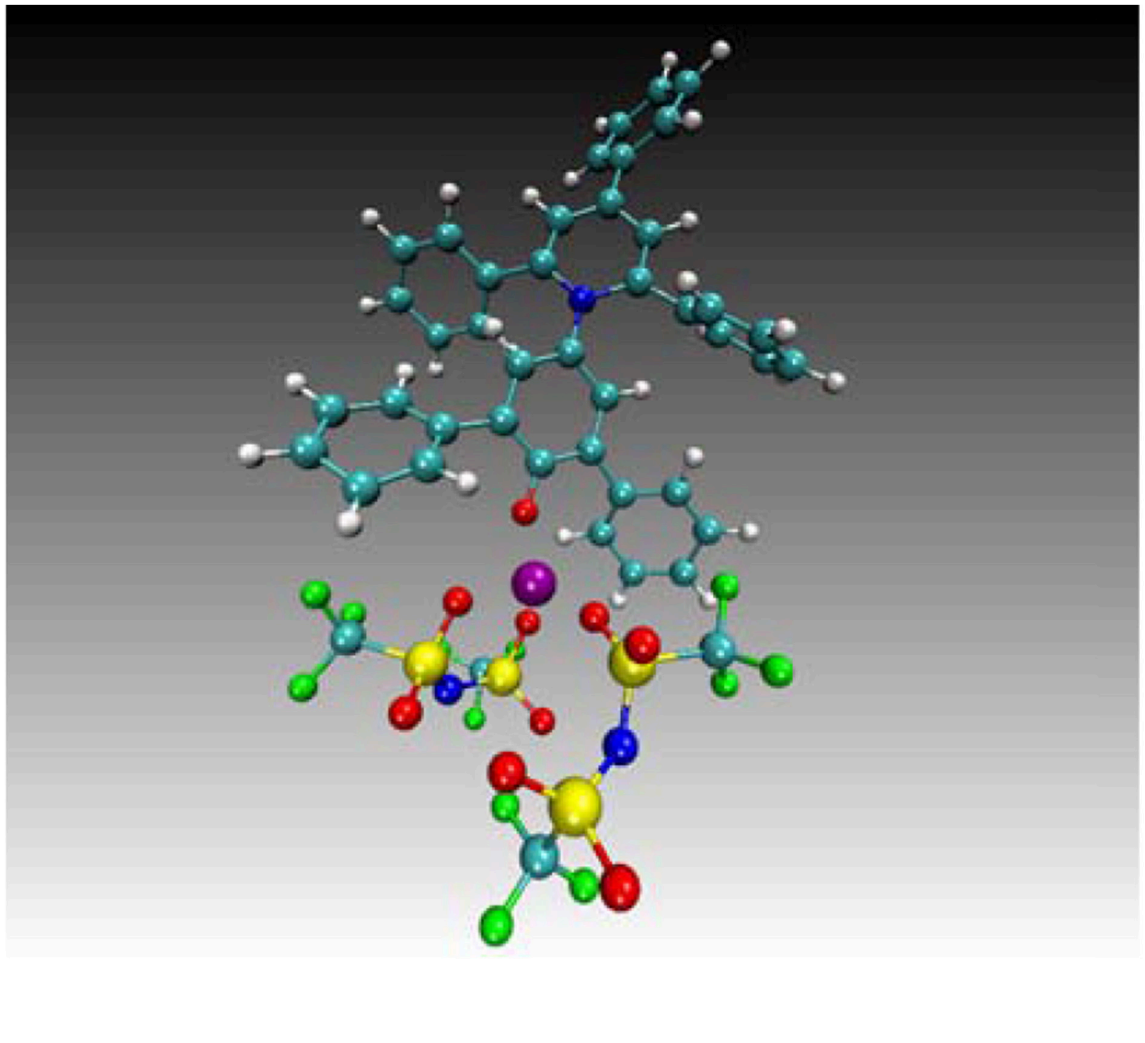
Typical configuration predicted for the dye—Li^+^ complex, showing additional coordination of three oxygen atoms provided by two TFSI molecules, taken from the [Li(G4)]TFSI liquid simulation. Remainder of the liquid not shown for clarity. Cyan = carbon, white = hydrogen, blue = nitrogen, red = oxygen, green = fluorine, yellow = sulfur and purple = Li^+^ (Eyckens et al., 2016b).

An unexpected result is the high α values for the SILs consisting of all glymes. This is attributed to the Lewis acidity of the chelated lithium cation, which is acting as a surrogate hydrogen bond. This phenomenon was confirmed by molecular dynamics, simulating the interaction between the chelated lithium cation of [Li(G4)]TFSI and Reichardt's dye (Figure 5) (Eyckens et al., 2016b).

The α values of the LiBETI derivatives of SILs are lower than those with the TFSI^−^ anion. This suggests there may be some greater attenuation of the cation in the presence of BETI^−^, or possibly the relative increase in size of BETI^−^ from TFSI^−^ (pentafluoroethyl vs. trifluoromethyl) suppressing the interaction of cation and dye. Note also, the determination of the α values of the by Dolan et al. (2016) were performed using Burgess' dye rather than Reichardt's, and all SILs exhibit lower values than those reported by others (Eyckens et al., 2016b; Black et al., 2018).

The Kamlet-Taft parameters for a traditional, imidazolium-based ionic liquid, [Bmim]TFSI (1-butyl-3-methylimidazolium *bis*(trifluoromethylsulfonyl)imide) have been included here to give context to the values of the SILs. The α value for this IL is a little under half of that of the SILs with the same anion, which may be due to the diffusion of the positive charge around the imidazolium ring, the acidic hydrogens or a combination of both (Eyckens et al., 2016b). In any event, the α value suffers from the absence of the hard lithium cation.

#### β-Values

The β values (hydrogen bond accepting ability) of the solvents also demonstrated the effect of chelation of lithium by the glyme/glycol units, in that the observed values of the SILs are comparatively lower than those of the glyme solvents on their own. This reduced hydrogen bond accepting ability of the solvents is caused by the electrons of the ethereal oxygens being engaged with the lithium-glyme chelate, and therefore limited for other hydrogen bonding entities. This is true across all SILs from all reports.

A similar value is exhibited by [Bmim]TFSI, though slightly lower. The hydrogen bond accepting characteristic of this IL is due to the anion. In the case of the SILs the accepting ability of the anion is bolstered with that of the chelating glyme, giving the slightly increased β values.

#### π-Values

Finally, the π^*^ (pi-star) values (polarity/polarizability) are the ability of the solvent to stabilize charge or become polarized. It would then follow that ionic compounds, like SILs, should have higher values than the solvating agents themselves. This observation is consistent across all reports with the introduction of either LiTFSI or LiBETI increasing the π^*^ values by about a third when compared to the glymes. A high π^*^ value is also observed for [Bmim]TFSI, which is typical for traditional ILs (Lee et al., 2008; Padró and Reta, 2016).

#### E_T_(30) and $E_{T}^{N}$–Solvent Polarity and Normalized Solvent Polarity

The determination of the E_T_(30) and the $E_{T}^{N}$ (solvent polarity and normalized solvent polarity, respectively) does not require any further measurements, only calculations using the data already obtained. The solvent polarity, E_T_(30), is referred to as such due to the betaine (Reichardt's dye) in the original report simply being the 30th dye investigated. This parameter is calculated using the maximum absorption of Reichardt's dye in the solvent which is then substituted into Equation 1 (Reichardt, 1994; Lee et al., 2008).

(1) $$\begin{array}{c} E_{T}\left(30\right)\left(kcal\;mol^{-1}\right)=\frac{28591}{v_{\max}\left(nm\right)} \end{array}$$

The E_T_(30) gives no real context to the polarity of the solvent, so it is therefore necessary to normalize this value on a scale of trimethylsilane (TMS, least polar, ${\text{E}}_{\text{T}}^{\text{N}}=0.000$) to water (most polar, ${\text{E}}_{\text{T}}^{\text{N}}=1.000$). This is accomplished using Equation 2 (Reichardt, 1994; Lee et al., 2008).

(2) $$\begin{array}{c} E_{T}^{N}=\frac{E_{T}\left(solvent\right)-E_{T}\left(TMS\right)}{E_{T}\left(water\right)-E_{T}\left(TMS\right)}=\frac{E_{T}\left(solvent\right)-30.7}{32.4} \end{array}$$

The results of these calculations are summarized in Table 3 (Eyckens et al., 2016b).

**Table 3:** The E_T_(30) (solvent polarity) and ${\text{E}}_{\text{T}}^{\text{N}}$ (normalized solvent polarity) of solvents (Eyckens et al., 2016b).

**Entry**	**Solvent**	**E_**T**_(30)**	**${\text{E}}_{\text{T}}^{\text{N}}$**
1	Water	63.1	1.000
2	[Li(G3)]TFSI	64.0	1.028
3	[Li(G4)]TFSI	64.2	1.033
4	Triglyme	40.4	0.301
5	Tetraglyme	39.9	0.284
6	[Bmim]TFSI	49.8	0.590

The ${\text{E}}_{\text{T}}^{\text{N}}$ gives a relative scale of the polarity of solvents compared to water as the most polar, ${\text{E}}_{\text{T}}^{\text{N}}$ = 1.000 (Entry 1, Table 3). [Li(G3)]TFSI and [Li(G4)]TFSI have ${\text{E}}_{\text{T}}^{\text{N}}$ values of 1.028 and 1.033 (Entry 2 & 3, Table 3), greater than that of water. Given the nature of ionic liquids comprising entirely of ions, it is not unexpected that they would have a high degree of polarity. This is due to the full cationic and anionic charges present in the IL, rather than the dipole moment experienced by water. The tri- and tetraglyme compounds are both around a third of the polarity of water with ${\text{E}}_{\text{T}}^{\text{N}}$ values of 0.301 and 0.284, respectively (Entry 4 & 5, Table 3). These ethereal solvents are expected to have a degree of polarity associated with them, due to the electronegativity of the oxygen atoms, and decreased ${\text{E}}_{\text{T}}^{\text{N}}$ values compared to the solvate ILs is due to the absence of ions. [Bmim]TFSI has a markedly reduced ${\text{E}}_{\text{T}}^{\text{N}}$ value compared to the solvate ILs, with 0.590 (Entry 6, Table 3), which is consistent with the higher α and β values of the solvate ILs.

### NMR Studies

Investigations into the electronic effects on the chelation of the lithium cation have been conducted by Black et al. using nuclear magnetic resonance (NMR) spectroscopy of ^7^Li, ^17^O, ^1^H and ^13^C nuclei (Black et al., 2018).

When examining ^7^Li in DMOS-*d*_6_, there is a reduction in chemical shift (moving to the right) of the signal from LiTFSI compared to the reference (9.7 mol kg^−1^ LiCl in H_2_O) (Black et al., 2018). This is due to the electron donation of the TFSI^−^ anion to the lithium cation. The introduction of the G1 and G2 glyme solvents initially results in the largest change (upfield) in ppm of the ^7^Li signal. This is attributed to the additive effect of the electron donation of the ethereal oxygens and the anion. This is true for both concentrations examined; 0.025 lithium atoms per oxygen atom, and 0.25 lithium atoms per oxygen atom.

Interestingly, the combination of the G3 and G4 solvents with LiTFSI demonstrated a less significant reduction in chemical shift, compared to that of the smaller glyme solvents. This is consistent with the chelation of the cation by the glyme having a greater effect, which may in part be due to the resulting steric bulk limiting anion-cation interaction.

A similar result is observed in the case of ^17^O NMR, though only the lesser concentration of LiTFSI in glyme was able to accurately be examined. The larger concentration (0.25 lithium per oxygen) resulted in only a broad signal that was unable to be deconvoluted. Nonetheless, the lesser concentration gives a good indication of the trend that is likely occurring in both scenarios.

With the introduction of the LiTFSI salt, the ethereal oxygen signals exhibit an upfield shift due to the chelation of the cation, resulting in an electron-poor oxygen atom (Peng et al., 2016). While this effect is initially counterintuitive, it has been demonstrated previously in aliphatic ether systems, that withdrawal of electron density from the oxygen results in a more shielded system, manifesting as a lower chemical shift (Béraldin et al., 1982).

A point of difference is observed between the terminal oxygen atoms (closest to the methyl group) and those of the ethylene linker, where the former exhibit a smaller change in chemical shift in the presence of the lithium cation. This can be ascribed to the lower degree of coordination of lithium by the terminal oxygens and this effect is exacerbated with larger glyme molecules, as there are more “non-terminal” oxygens available for chelating.

In determining the changes in the ^1^H NMR shifts of the glyme compounds, there is a trend of a decrease in chemical shift with the introduction of LiTFSI. Further, a greater change in ppm of the methyl protons than the signals corresponding to the methylene protons (relative to the pure glyme values) is observed.

This is again counterintuitive, as a coordination of the oxygen atoms to lithium should draw on electrons, shifting the signals of the relative protons downfield, and the draw of two oxygen atoms should have a greater effect on methylene protons than methyl. The effect is credited to interaction of protons with the TFSI^−^ anion, effectively negating the electron withdrawing effect by donating electron density to these protons.

The more withdrawn the protons, as is the case with those of the methylene, the greater the susceptibility to anion interaction. These two processes effectively cancel each other out, resulting in the negligible change in shift for the methylene protons. In the case of the methyl protons however, the balance between effects is not as evenly matched, resulting in a more prominent change in signal resonance, albeit a small one. It may also be possible that the electron withdrawn nature of the protons with the chelation of the lithium cation contributes to some degree to the unanticipatedly large α value exhibited by the SILs.

Notably, this phenomenon is somewhat reversed when exploring the effects through ^13^C NMR. The carbons of the methyl groups show no significant change in chemical shift in the solvate ILs with respect to the pure glyme. For the methylene carbons however, there is a decrease in shift resulting from the balance of interaction with both the lithium cation and TFSI^−^ anion.

This investigation clearly demonstrates the significant role played by the anion on the electronics of the SIL complex. These interactions between glyme ligand and cation become more significant with increasing glyme length, causing a reduction in cation-anion interaction.

The correlation of these observations with molecular modeling would potentially compliment these studies and provide potential insight into the intricacies of this system.

#### Gutmann Acceptor Number

Given the high α values of the SILs and the confirmation of this effect with molecular dynamics, the *pseudo*-hydrogen bonding may be attributed to Lewis acidity, which can be examined using a phosphorous-based probe, and it analysis *via* ^31^P NMR. The Gutmann Acceptor Number is a measurement of the Lewis and measures the interaction of the desired solvent and a Lewis base, triethylphosphine oxide (Et_3_PO) in a 3:1 ratio (solvent:base) dissolved in deuterated benzene (Mayer et al., 1975; Gutmann, 1976).

**Figure 6:**
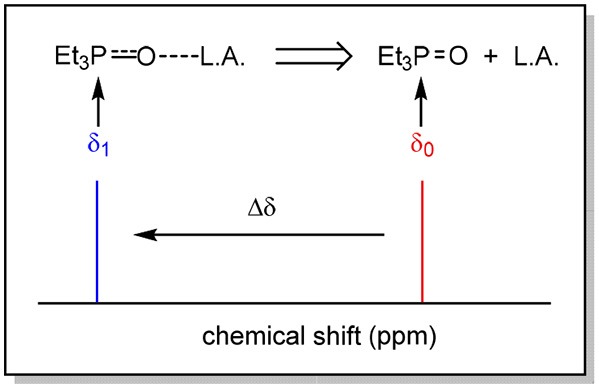
Representation of the effect of a Lewis acid on the chemical shift of phosphorous in the ^31^P NMR spectrum.

The Acceptor Number (AN) is determined by monitoring the shift of the phosphorous atom when introduced to a Lewis acid by polarization of the P = O bond. The greater the strength of the Lewis acid, the more electron withdrawn the phosphorous atom becomes, shifting the resonance of that atom in the ^31^P NMR spectra downfield (to the left, Figure 6).

To accurately ascertain the degree of shift of the phosphorous resonance, the Lewis base must first be measured in relation to a non-Lewis acidic solvent, as a control measurement (*n*-heptane was used in this case).

The Acceptor Numbers of solvents are summarized in Table 4 (Eyckens et al., 2016a).

**Table 4:** Acceptor Number (AN) of Solvate ILs (Eyckens et al., 2016a).

**Entry**	**Lewis acid**	**Et**_****3****_**PO**		**AN**
		**δ(^**31**^P)**	**Δδ(^**31**^P)**	
1	*n*-heptane	46.66	0.00	0.00
2	LiTFSI (salt)	64.48	17.82	41.84
3	[Li(G3)]TFSI	57.96	11.30	26.53
4	[Li(G4)]TFSI	57.96	11.30	26.53
5	Triglyme	46.76	0.10	0.24
6	Tetraglyme	46.75	0.09	0.21
7	[Bmim]TFSI	51.68, 47.94	5.07, 1.30	11.90, 3.10

**Figure 7:**
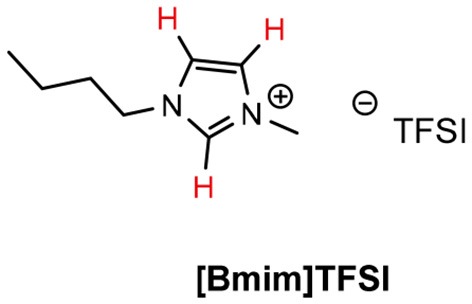
Acidic protons of [Bmim]TFSI shown in red.

It was of interest to determine the AN of the naked LiTFSI salt, to quantify the effect chelation had on the lithium cation. The salt exhibited an Acceptor Number of 41.84 (Entry 3, Table 4), almost double that of the solvate ILs. Lithium has been shown to be a strong Lewis acid, often used as an additive for organic reactions (Springer et al., 1999), so in the non-chelated form, it is logical to expect this increase in Lewis acidity. [Li(G3)]TFSI and [Li(G4)]TFSI demonstrated identical AN with 26.53 each (Entry 4 & 5, Table 4). This represents the effect of chelation, as the lithium cation is not as free to interact with the Lewis base, the effect may be due to a combination of electronic and steric effects. The tri- and tetraglyme molecules showed the expected very low AN with 0.24 and 0.21, respectively (Entry 6 & 7, Table 4). The negligible Lewis acidity exhibited by these compounds, is to be expected due to the absence of electron-accepting functional groups. [Bmim]TFSI shows reduced Lewis acidity when compared to the solvate ILs with AN values of 11.90 and 3.10 (Entry 8, Table 4). The two chemical shifts are due to the nature of the cation, as Lewis acids are typically metal-based and the amine cation is not as efficient at accepting electrons. Presumably, the observed Lewis acidity of [Bmim]TFSI is dominated by hydrogen-bonding effects between the phosphine oxide and the acidic hydrogen atom at C2, and the pair at C4/C5 on the imidazolium ring (Figure 7), giving the two values.

In summary, solvate ionic liquids represent a unique class of liquids which possess interesting Lewis Acidic properties, with the typical features of ionic liquids being negligible vapor pressure and high polarity. These unique properties, in addition to their cryogenic melting points and absence of acidic protons, opens the potential of these liquids to be used as media for organic chemical transformations which are typically inaccessible using imidazolium-derived or protic ionic liquids. The first reports of SILs as reaction media and their effect on reaction outcome are reported in the following section.

## Applications in Synthetic Chemistry

Ionic liquids as reaction mediums for organic transformations are already well-established in the literature, with many reviews available detailing their use (Welton, 1999; Wasserscheid and Keim, 2000; Hallett and Welton, 2011; Qureshi et al., 2014). In comparison, solvate ILs have seen very little representation in the literature, though the examples that do exist are reported here.

### Organocatalysis

Juaristi and co-workers report a highly efficient, and stereoselective asymmetric aldol reaction when using (*S*)-proline in conjunction with solvate ionic liquids (Table 5, below) (Obregón-Zúñiga et al., 2017). The solvates explored are both the [Li(G3)]TFSI and [Li(G4)]TFSI, as well as the perchlorate versions; the [Li(G3)]ClO_4_ and [Li(G4)]ClO_4_. It should be noted that [Li(G3)]ClO_4_ is reported as a solid (mp 103°C) (Ueno et al., 2012, 2015; Mandai et al., 2014; Obregón-Zúñiga et al., 2017), and [Li(G4)]ClO_4_ is considered a concentrated solution, rather than a solvate ionic liquid, due to the excess of free glyme (~20%) in the system (Ueno et al., 2015).

**Table 5:** Evaluation of various SILs and blanks in the model aldol reaction (Obregón-Zúñiga et al., 2017).

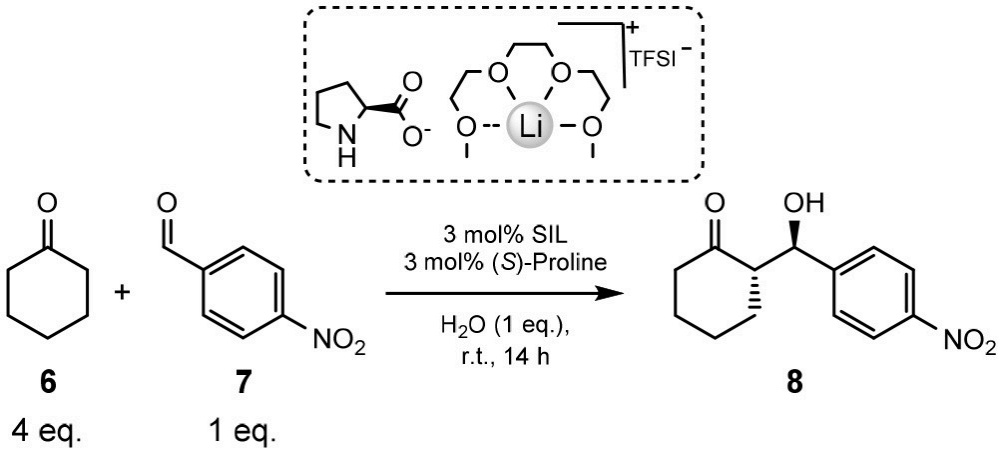				
**Entry**	**SIL**	**Yield (%)**^a^	***dr* (*anti:syn*)^b^**	***er* (*anti*)^c^**
1	[Li(G3)]TFSI	94	94:6	98:2
2	[Li(G4)]TFSI	96	90:10	96:4
3	[Li(G3)]ClO_4_	84	93:7	98:2
4	[Li(G4)]ClO_4_	87	89:11	94:6
5^d^	-	18	64:36	88:12
6	-	67	82:18	92:8

a *Isolated yield*.

b *Determined by ^1^H NMR from the crude reaction*.

c *Determined by HPLC with chiral stationary phase*.

d *No water added, 24 h of reaction*.

Nevertheless, these particulars are of little consequence due to the greater performance exhibited by the TFSI^−^ anion varietals compared to the ${\text{ClO}}_{4}^{-}$ versions (Table 5). It was found that an equimolar mixture of SIL and (*S*)-proline employed at 3 mol% with 1 equivalent of water was the optimal conditions for the aldol reaction to take place, at ambient temperature for 14 h (Obregón-Zúñiga et al., 2017).

Employing [Li(G3)]TFSI with (*S*)-proline exhibits an excellent yield (94%), *dr* (94:6) and *er* (98:2) in the aldol reaction between cyclohexanone **6** and 4-nitrobenzaldehyde **7** (Entry 1, Table 5). The yield is slightly improved (96%) with the use of the tetraglyme counterpart, [Li(G4)]TFSI, though both the diastereomeric ratio and enantiomeric ratio suffer (Entry 2, Table 5). this leads to the conclusion that, despite a slight reduction yield, [Li(G3)]TFSI is the better performing SIL.

[Li(G3)]ClO_4_ gives the lowest yield of all the additives (84%, Entry 3, Table 5), though does see an improvement in *dr* (93:7) when compared to [Li(G4)]TFSI (90:10), and the same *er* as [Li(G3)]TFSI (98:2). This may suggest some conformational benefit of the triglyme over the tetraglyme, as [Li(G4)]ClO_4_ lead to worse *dr* and er, but a slight increase in yield (87%, Entry 4, Table 5).

To give these results context, the reaction was repeated without the addition of any SILs or water (Entry 5, Table 5). This instance shows, as expected, the lowest yield (18%), diastereomeric and enantiomeric ratios (64:36 and 88:12, respectively), and an increase in reaction time to 24 h. Immediate improvement is observed with the addition of 1 equivalent of water, with higher yield (67%) and better *dr* and *er* (82:18 and 92:8, respectively, Entry 6, Table 5).

The stereoselective advantage of [Li(G3)]TFSI in this aldol reaction is proposed to be the formation of a supramolecular aggregate between the SIL and (*S*)-proline (Obregón-Zúñiga et al., 2017).

This structure is supported by both ^7^Li NMR and IR spectroscopic methods (Obregón-Zúñiga et al., 2017), and the transition state of the supramolecular ensemble containing ketone, aldehyde, water and the (*S*)-proline-[Li(G3)]TFSI complex was also modeled (Obregón-Zúñiga et al., 2017). The complex shows the 1 equivalent of water acting as a bridgehead between the carbonyls of the ketone and aldehyde.

The effect of [Li(G3)]TFSI in this aggregation is observed to be specific for SILs, as a comparison is conducted with a traditional IL; [Bmim]PF_6_ and one without any ionic species present (Obregón-Zúñiga et al., 2017). This is also explored in the context of electron withdrawn, neutral and electron donating aldehydes with cyclohexanone.

In every case examined, [Li(G3)]TFSI was the best performing example with higher yields and diastereomeric and enantiomeric ratios. The best results obtained of the three types of aromatic aldehydes were those that are electron withdrawn, as may be expected. Further demonstrating the requirement for the SIL complex, is the poor result obtained when using the lithium carboxylate of (S)-proline as the catalyst, or just the LiTFSI.

This work demonstrates the advantage of SILs in an organic transformation, over a traditional IL ([Bmim]PF_6_), [Li(G3)]ClO_4_ or [Li(G4)]ClO_4_. The latter proves the requirement for the solvation of the lithium cation, as it has been defined as a concentrated solution (Ueno et al., 2015).

### Diels-Alder Reactions

To utilize the Lewis acidity of [Li(G3)]TFSI and [Li(G4)]TFSI, both SILs were assessed in their application as solvents for Diels–Alder reactions (Eyckens et al., 2016a). The [4+2] cycloadditions of a series of reactants are summarized in Table 6.

**Table 6:** Diels–Alder reactions in solvate ionic liquids (Eyckens et al., 2016a).

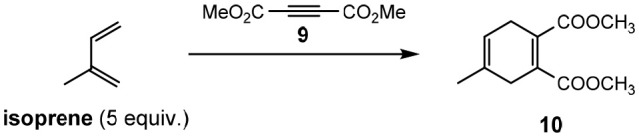				
**Entry**	**Solvent**	**Time**	**Temp. (°C)^a^**	**Yield (%)^b^**
1	5.0 M LPDE	7 h	r.t.	94^c^
2	[Bmim] PF_6_	2 h	80^d^	96^e^
3	[Li(G3)]TFSI	30 min	100	73
4	[Li(G4)]TFSI	30 min	100	81

a *Microwave irradiation*.

b *Isolated yield*.

c *Reported yield (Grieco et al., 1990)*.

d *No microwave irradiation*.

e *Reported yield (Earle et al., 1999)*.

A similar solvent, 5.0 M Lithium Perchlorate in Diethyl Ether (5.0 M LPDE) saw good application for electrocyclization reactions in the 1990s to 2000s (Grieco et al., 1990; Forman and Dailey, 1991; Grieco and Moher, 1993; Heydari, 2002), but soon fell out of favor due to a lack of handleability and stability of the ${\text{ClO}}_{4}^{-}$ anion at elevated temperatures. It has been included here as the closest point of comparison to the SILs in terms of a reaction medium. The limitation of not being able to heat 5.0 M LPDE due to potential explosion is not relevant to the solvate ILs, and thus this fact was exploited during reaction optimization.

The Diels–Alder reaction between isoprene and **9** is reported to proceed well at ambient temperature in 5.0 M LPDE (94% in 7 h, Entry 1, Table 6). It has also been shown to be successful in [Bmim]PF_6_, with the addition of heat (96% in 2 h, Entry 2, Table 6). The reported conditions for the Diels–Alder reaction in ionic liquids use 5 equivalents of the diene, and this was kept consistent in this study to allow for greater comparison. However, isoprene was observed to be only sparingly soluble in the solvate ILs, forming a biphasic solution when added to the reaction vessel. This suggests that a much lower effective concentration was required to complete these reactions than in the instances of other ionic liquids, meaning fewer equivalents may have been able to be used to produce the same result.

Performing the reaction for 30 min at 100°C, using microwave irradiation (Entry 3 & 4, Table 6) gave good yields for both [Li(G3)]TFSI and [Li(G4)]TFSI (73 and 81%, respectively). This demonstrated the advantages of being able to heat these solvate ILs without limitation (such as those outlined for 5.0 M LPDE) as part of an optimization process.

The Diels–Alder reaction between isoprene and dimethyl maleate (**11a**) or dimethyl fumarate (**11b**) was also investigated. Previous reports (Renninger and Mcphee, 2008) of the Diels–Alder reaction between dimethyl maleate (**11a**) and isoprene required the use of extremely high temperatures to obtain good yields (cf. 195°C and 66%, respectively). Attempting the reaction in a molecular solvent (chloroform) proved the difficult nature of this reaction when only traces of the desired product **12** obtained (Entry 1, Table 7) despite relatively high temperatures. Employing [Li(G3)]TFSI and [Li(G4)]TFSI (Entry 2 & 3, Table 7) showed greater promise, with isolated yields of 73 and 21%, respectively. Despite this, it was found that the isolated product was **12b**, possessing the *trans* configuration of the esters and not the expected *cis* transfiguration of **12a**. This is unusual as stereochemistry is typically retained throughout the Diels-Alder reaction, and it was concluded that while the product **12a** may form initially, it is possible for this product to convert to the more thermodynamically stable **12b**.

**Table 7:** Diels–Alder reactions in solvate ionic liquids (Eyckens et al., 2016a).

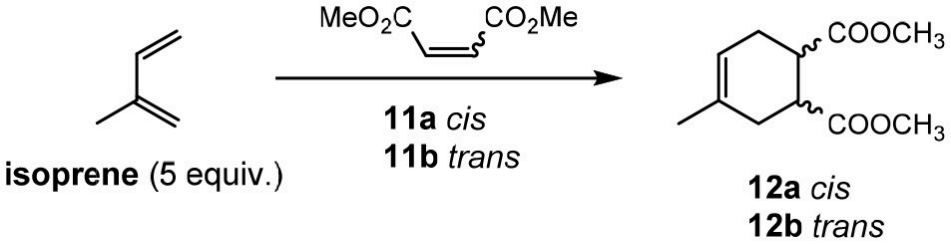					
**Entry**	**Reactant**	**Solvent**	**Time**	**Temp. (°C)^a^**	**Yield (%)^b^**
1	**11a**	CHCl_3_	20 min	100	<5
2	**11a**	[Li(G3)]TFSI	20 min	100	73^c^
3	**11a**	[Li(G4)]TFSI	20 min	100	21^c^
4	**11a**	[Li(G3)]TFSI	16 h	r.t.	0
5	**11b**	CHCl_3_	20 min	100	45
6	**11b**	[Li(G3)]TFSI	20 min	100	74
7	**11b**	[Li(G4)]TFSI	20 min	100	65

a *Microwave irradiation*.

b *Isolated yield*.

c *Only trans product isolated*.

This epimerization is presumed to be due to the higher temperature employed in this reaction. The dramatic reduction in yield in [Li(G4)]TFSI is unknown, but may be due to a slightly different solubility of isoprene in this IL compared to the [Li(G3)]TFSI variation.

This reaction was then repeated with **11a** at room temperature in [Li(G3)]TFSI overnight (Entry 4, Table 7) to minimize possible epimerization. Unfortunately, the reaction was unsuccessful and only starting material **11a** was isolated.

Dimethyl fumurate (**11b**) was then employed as the dienophile, and a much less pronounced improvement in yield was obtained in the solvate ILs, when compared to the molecular solvent (Entry 5, Table 7 *vs*. Entry 6 & 7, Table 7). When compared to previous reports (Lee et al., 2009) however (*cf*. 36 h, 70°C), the reaction required considerably less time to occur.

### [2+2] Cycloaddition Cascade Formation of Dienes

Another electrocyclisation reaction attempted in the solvate ILs was the [2+2] reaction of dimethyl ketene (**13**) (generated *in situ* from isobutyryl chloride) and (*E*)-cinnamaldehyde (**14**) giving **15** after CO_2_ extrusion (Eyckens et al., 2016a). This reaction is reported not to proceed in the absence of LiClO_4_ and has been reported in 5.0 M LPDE, therefore is an obvious choice to compare the application of the solvate ILs.

Conducting the reaction in chloroform resulted in trace amounts of product formed, as was expected (Entry 1, Table 8). When repeating the literature conditions (Arrastia and Cossío, 1996), the high yield (95%) originally reported in 5.0 M LPDE was unable to be reproduced (Entry 2, Table 8). Employing freshly prepared 5.0 M LPDE, from extensively dried LiClO_4_ and diethyl ether, saw highly variable yields at best. The yield of **15** in 5.0 M LPDE varied between 5–40%, with <20% being typical for repeated attempts. Despite the complete consumption of the aldehyde **14**, a complex mixture of products was typically observed in the ^1^H NMR spectra of the crude mixture.

**Table 8:** Comparison of solvents in [2+2] cascade formation of dienes (Eyckens et al., 2016a).

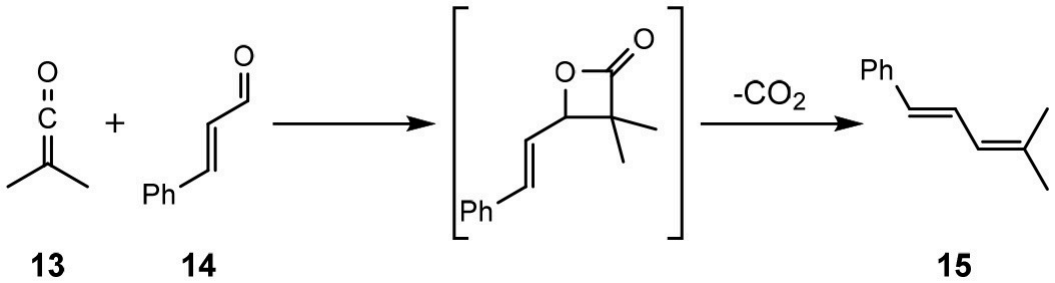				
**Entry**	**Solvent**	**Time (h)**	**Temp. (°C)**	**Yield (%)^a^**
1	CHCl_3_	6	r.t.	<5
2	5.0 M LPDE	6	r.t.	~20
3	[Li(G3)]TFSI	6	r.t.	56
4	[Li(G4)]TFSI	6	r.t.	41
6^b^	[Li(G3)]TFSI	6	r.t.	60
7	[Li(G3)]TFSI	6^c^	80	70

a *Isolated yield*.

b *Activated molecular sieves (100 mg) were used throughout the reaction*.

c *The reaction mixture was heated for the finally hour of the specified time*.

The complete consumption of the aldehyde **14** was also observed in the ^1^H NMR spectra of crude mixtures of the reactions carried out in [Li(G3)]TFSI and [Li(G4)]TFSI, though the isolated yields were greatly improved; 56% and 41%, respectively (Entry 3 & 4, Table 8). It was hypothesized that atmospheric water may be affecting the reaction outcomes. In an attempt to remedy this and improve yields, 4 Å molecular sieves were added to the reaction in [Li(G4)]TFSI to remove adventitious water, improving the yield to 60% (Entry 6, Table 8). Further to this, heating for the final hour of the reaction to 80°C in [Li(G3)]TFSI increased the yield to 70% (Entry 7, Table 8).

These reactions highlight the ease with which these SILs can be handled. The use of molecular sieves and heated using 5.0 M LPDE is not possible, due to precipitation of lithium perchlorate if taken out of inert atmosphere, and again the limitations of heating in this system.

### α-Aminophosphonates

α-Aminophosphonates are small, phosphorous containing molecules, structurally analogous to naturally occurring α-amino acids.

Replacement of the carbonyl component of the amino acid with phosphorous has been shown to inhibit enzymes of receptors to which the natural amino acids bind (Cherkasov and Galkin, 1998). This lays the foundation for α-aminophosphonates to have medicinal or therapeutic applications.

These compounds are typically accessed *via* the Kabachnik-Fields reaction, discovered independently by both Kabachnik and Ya (1952) and Fields (1952). The reaction proceeds through condensing an amine with an aldehyde (either *in situ* or preformed), before reaction with a phosphonate.

Conducting this reaction in SILs at room temperature for 5 min demonstrated the broad scope of reactants suitable in these conditions. The advantage of SILs is evident as other reports of the synthesis of these molecules utilize increased reaction time [up to 7 days (Pettersen et al., 2006)], temperature (Guo et al., 2015), the use of boutique catalysts (Ambica et al., 2008; de Noronha et al., 2011; Heo et al., 2012; Li et al., 2016) or combinations thereof.

The success of the Kabachnik-Fields reaction in solvate ILs is demonstrated through the application of a range of substituted aldehydes and anilines with diphenyl phosphite (Table 9), (Eyckens and Henderson, 2017).

**Table 9:** Scoping of aniline derivatives to produce α-aminophosphonates in SILs (Eyckens and Henderson, 2017).

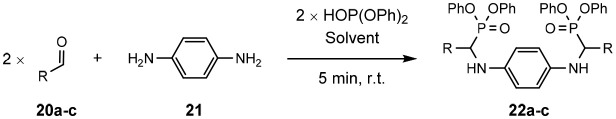								
**Entry**	**Solvent**	**Aniline**	**${\text{R}}_{1}^{\text{a}}$**	**Yield (%)^b^**	**Entry**	**Aldehyde**	**R${\text{R}}_{2}^{\text{a}}$**	**Yield (%)^b^**
1	[Li(G3)]TFSI	**16a**	4-NO_2_	64	15	**17h**	4-Br	90
2	[Li(G4)]TFSI			25	16			91
3	[Li(G3)]TFSI	**16b**	4-OH	82	17	**17i**	4-Me	90
4	[Li(G4)]TFSI			92	18			86
5	[Li(G3)]TFSI	**16c**	4-F	84	19	**17j**	4-NO_2_	69
6	[Li(G4)]TFSI			68	20			59^c^
7	[Li(G3)]TFSI	**16d**	4-Cl	96	21	**17k**	4-F	77
8	[Li(G4)]TFSI			96	22			78
9	[Li(G3)]TFSI	**16e**	3-Cl	83	23	**17l**	2-OH	84
10	[Li(G4)]TFSI			59	24			76
11	[Li(G3)]TFSI	**16f**	3-CF_3_	86	25	**17m**	3,4-Cl	74
12	[Li(G4)]TFSI			81	26			79
13	[Li(G3)]TFSI	**16g**	3,5-CF_3_	54				
14	[Li(G4)]TFSI			60				

*^a^Alternate R group is hydrogen*.

b *Isolated yield*.

c *This material contained α-hydroxyphosphonate (17% by 1H NMR) resulting from direct attack of the phosphite on 4-nitrobenzaldehyde*.

The production of a range of α-aminophosphonates with various electron withdrawing or donating functional groups on either the aniline or benzaldehyde was rapidly achieved, with overall excellent yields (Table 9). It was observed that, in general, [Li(G3)]TFSI proved to be the better performing solvent of the two SILs in most cases. This is consistent with previous findings when using these SILs as reaction solvents, although the difference between the two solvents is generally quite small.

In addition to the production of these *mono*-α-aminophosphonates, is the synthesis of *bis*-versions, utilizing arylenediamines (Table 10).

**Table 10:** Scoping of *bis*-α-aminophosphonates (Eyckens and Henderson, 2017).

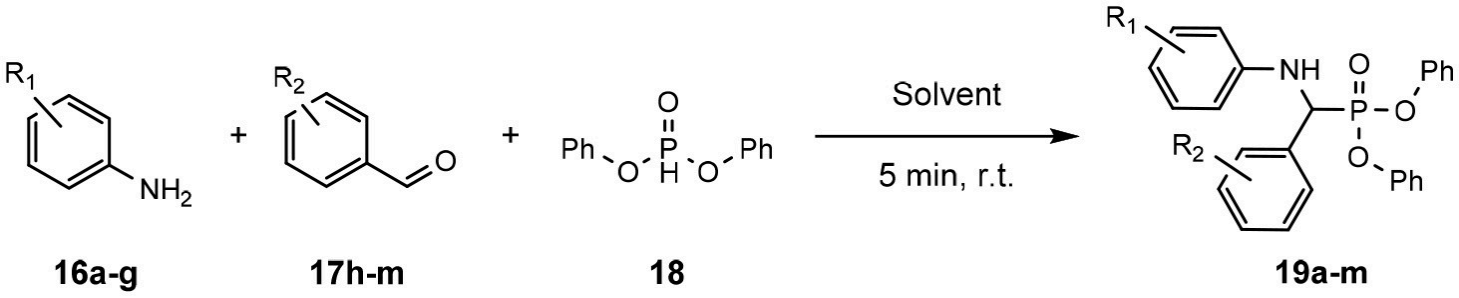				
**Entry**	**Product**	**R**	**Solvent**	**Yield (%)^a^**
1	**22a**	Ph	[Li(G3)]TFSI	64
2			[Li(G4)]TFSI	52
3	**22b**	4-BrPh	[Li(G3)]TFSI	36
4			[Li(G4)]TFSI	65
5	**22c**	4-NO_2_Ph	[Li(G3)]TFSI	18
6			[Li(G4)]TFSI	33

a *Isolated yield*.

The synthesis of *bis*-α-aminophosphonates in SILs is demonstrated with good success (Table 10), maintaining the very concise reaction time and good substrate tolerance.

Unlike the earlier trend of [Li(G3)]TFSI outperforming [Li(G4)]TFSI in the production of *mono*-α-aminophosphonates, there seems a reversal in this trend when introducing substituted aldehydes into the *bis*-versions of these α-aminophosphonates **22b-c**.

**Scheme 4:**
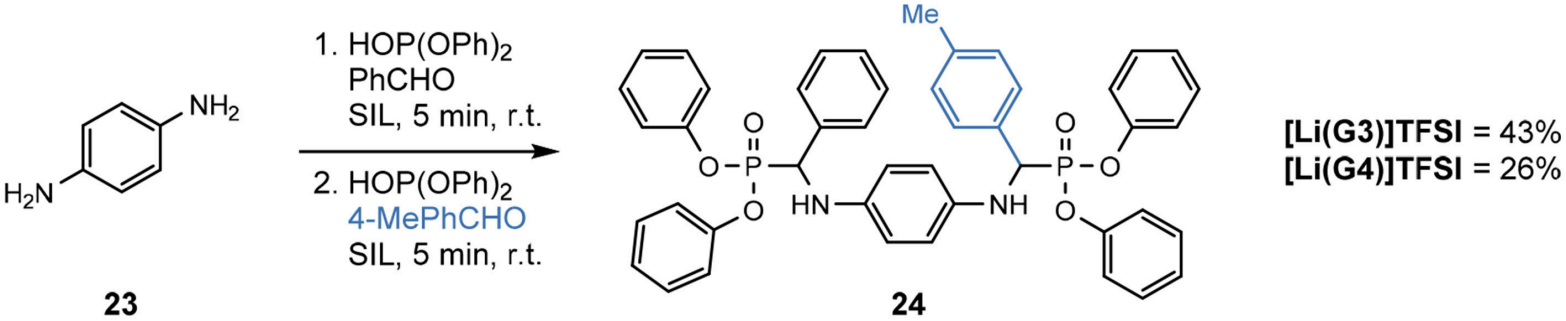
Synthesis of a non-C_2_-symmetric *bis*-α-aminophosphonate 24 from *p*-phenylenediamine and incorporating different aldehydes in the SILs (Eyckens and Henderson, 2017).

In addition to the successful synthesis of *bis*-versions of α-aminophosphonates, the efficacy of producing a non-C_2_-symmetric *bis*-α-aminophosphonate using two different aldehydes was investigated (Scheme 4).

The non-C_2_-symmetric compound **24** was synthesized in good yields in both SILs (43% in [Li(G3)]TFSI and 26% in [Li(G4)]TFSI isolated yields), in only 10 min of total reaction time at room temperature. Each aldehyde was reacted for 5 min in a two-step, one-pot process. The use of 4-tolualdehyde served as a simple ^1^H NMR handle to determine the incorporation of both aldehydes through comparison of integration ratios of the methyl signal and the protons attached to the tertiary carbon.

The use of SILs as solvents for organic reactions has shown great success, even in the limited number of reports available. The comparison to molecular solvents, other ionic liquids or even the use of catalysts has advocated strong advantage in the use of synthesis. The ability to access products of cycloaddition or condensation reactions using these solvents shows great versatility and utility.

## Conclusions and outlook

The physical parameters of solvate ionic liquids have been discussed here, as reported by a number of research groups. Consist findings of high α values (hydrogen bond donating) are recorded, despite the absence of acidic protons. This was largely attributed to the lithium cation acting as a surrogate hydrogen bond, which was confirmed by molecular dynamics simulations. The high α values were also consolidated by the determination of Lewis acidity (Gutmann Acceptor Number), revealing identical values for both SILs (AN = 26.5). This value was reduced in comparison to the naked LiTFSI salt and increased compared to that of the pure glymes.

The β values were reduced in comparison to the pure glyme solvents, anticipating the chelation of lithium by the ethereal oxygens, thereby limiting their availability for hydrogen bond accepting. This effect of chelation was also supported by NMR studies of all relevant nuclei. Finally, the π^*^ values demonstrated the SILs' polarizability which was expectedly high due to their ionic character, and consistent with traditional ionic liquids. The SILs were also observed to be as polar (if not slightly more so) as water.

The application of SILs to effect reaction outcomes was validated in conjunction with a proline organocatalyst with excellent results in the aldol reaction. This success was further echoed by the use as a solvent for Diels-Alder and [2+2] cycloaddition reactions, with good yields reported.

The application to a different type of reaction in the Kabachnik-Fields reaction further conveyed the advantage of SILs, rapidly accessing α-aminophosphonates in excellent yields at room temperature, with good scope and the ability to produce *bis*-versions (including non-C_2_-symmetric) of these compounds.

The foundational research explored here serves as a good basis for future investigations, and the use of these SILs as solvents for organic transformations is a field with huge potential. The potential to access molecules in less time, temperature or both is enticing and offers great advantage over previous methods.

## Author Contributions

All authors listed have made a substantial, direct and intellectual contribution to the work, and approved it for publication.

### Conflict of Interest Statement

The authors declare that the research was conducted in the absence of any commercial or financial relationships that could be construed as a potential conflict of interest.

## References

[B1] Abraham K. M. Jiang Z. Carroll B. (1997). Highly conductive PEO-like polymer electrolytes. Chem. Mater. 9, 1978–1988. 10.1021/cm970075a

[B2] Ambica K. S. Taneja S. C. Hundal M. S. Kapoor K. K. (2008). One-pot synthesis of α-aminophosphonates catalyzed by antimony trichloride adsorbed on alumina. Tetrahedron Lett. 49, 2208–2212. 10.1016/j.tetlet.2008.02.047

[B3] Angell C. A. Byrne N. Belieres J. P. (2007). Parallel developments in aprotic and protic ionic liquids, physical chemistry and applications. Acc. Chem. Res. 40, 1228–1236. 10.1021/ar7001842 17979250

[B4] Arrastia I. Cossío F. P. (1996). Tandem [2+2] cycloaddition-cycloreversion reactions in highly polar media: a convergent one-pot entry to substituted alkenes and dienes. Tetrahedron Lett. 37, 7143–7146. 10.1016/0040-4039(96)01562-6

[B5] Atilhan M. Aparicio S. (2014). Folding of graphene nanostructures driven by ionic liquids nanodroplets. J. Phys. Chem. C. 118, 21081–21091. 10.1021/jp502303q

[B6] Atta N. F. Abdel Gawad S. A. El-Ads E. H. El-Gohary A. R. M. Galal A. (2017). A new strategy for NADH sensing using ionic liquid crystals-carbon nanotubes/nano-magnetite composite platform. Sensors Actuators B Chem. 251, 65–73. 10.1016/j.snb.2017.05.026

[B7] Austen Angell C. Ansari Y. Zhao Z. (2012). Ionic liquids: past, present and future. Faraday Discuss. 154, 9–27. 10.1039/C1FD00112D 22455011

[B8] Béraldin M.-T. Vauthier E. Fliszár S. (1982). Charge distributions and chemical effects. XXVI. Relationships between nuclear magnetic resonance shifts and atomic charges for 17O nuclei in ethers and carbonyl compounds. Can. J. Chem. 60, 106–110. 10.1139/v82-020

[B9] Black J. J. Dolan A. Harper J. B. Aldous L. (2018). Kamlet–Taft solvent parameters, NMR spectroscopic analysis and thermoelectrochemistry of lithium–glyme solvate ionic liquids and their dilute solutions. Phys. Chem. Chem. Phys. 20, 16558–16567. 10.1039/C8CP02527D 29873357

[B10] Bordes É. Szala-Bilnik J. Pádua A. A. H. (2018). Exfoliation of graphene and fluorographene in molecular and ionic liquids. Faraday Discuss. 206, 61–75. 10.1039/C7FD00169J 28933474

[B11] Butler B. J. Harper J. B. (2018). The effect of the structure of the anion of an ionic liquid on the rate of reaction at a phosphorus centre. J. Phys. Org. Chem. 32:e3819 10.1002/poc.3819

[B12] Chaban V. V. Andreeva N. A. Fileti E. E. (2018). Graphene/ionic liquid ultracapacitors: does ionic size correlate with energy storage performance? New J. Chem. 42, 18409–18417. 10.1039/C8NJ04399J

[B13] Chaban V. V. Fileti E. E. (2015). Graphene exfoliation in ionic liquids: unified methodology. RSC Adv. 5, 81229–81234. 10.1039/C5RA16857K

[B14] Chaban V. V. Fileti E. E. Prezhdo O. V. (2017). Exfoliation of graphene in ionic liquids: pyridinium versus pyrrolidinium. J. Phys. Chem. C 121, 911–917. 10.1021/acs.jpcc.6b11003

[B15] Chee W. K. Lim H. N. Zainal Z. Huang N. M. Harrison I. Andou Y. (2016). Flexible graphene-based supercapacitors: a review. J. Phy. Chem. C. 120, 4153–4172. 10.1021/acs.jpcc.5b10187

[B16] Chen D. Zhuang X. Zhai J. Zheng Y. Lu H. Chen L. (2018). Preparation of highly sensitive Pt nanoparticles-carbon quantum dots/ionic liquid functionalized graphene oxide nanocomposites and application for H2O2 detection. Sens. Act. B: Chem. 255, 1500–1506. 10.1016/j.snb.2017.08.156

[B17] Cherkasov R. Galkin V. I. (1998). The Kabachnik-fields reaction: synthetic potential and the problem of the mechanism. Russian Chem. Rev. 67, 857–882. 10.1070/RC1998v067n10ABEH000421

[B18] Cook A. Ueno K. Watanabe M. Atkin R. Li H. (2017). Effect of variation in anion type and glyme length on the nanostructure of the solvate ionic liquid/graphite interface as a function of potential. J. Phy. Chem. C. 121, 15728–15734. 10.1021/acs.jpcc.7b03414

[B19] Crowhurst L. Falcone R. Lancaster N. L. Llopis-Mestre V. Welton T. (2006). Using kamlet–taft solvent descriptors to explain the reactivity of anionic nucleophiles in ionic liquids. J. Organ. Chem. 71, 8847–8853. 10.1021/jo0615302 17081015

[B20] de Noronha R. G. Romão C. C. Fernandes A. C. (2011). MoO2Cl2 as a novel catalyst for the synthesis of α-aminophosphonates. Catal. Commun. 12, 337–340. 10.1016/j.catcom.2010.10.005

[B21] Dolan D. A. Sherman D. A. Atkin R. Warr G. G. (2016). Kamlet–taft solvation parameters of solvate ionic liquids. ChemPhysChem. 17, 3096–3101. 10.1002/cphc.201600361 27337999

[B22] Earle J. M. McCormac P. B. Seddon K. R. (1999). Diels-Alder reactions in ionic liquids. A safe recyclable alternative to lithium perchlorate-diethyl ether mixtures. Green Chem. 1, 23–25. 10.1039/a808052f

[B23] Eyckens D. J. Champion M. E. Fox B. L. Yoganantharajah P. Gibert Y. Welton T. (2016a). Solvate ionic liquids as reaction media for electrocyclic transformations. Eur. J. Org. Chem. 2016, 913–917. 10.1002/ejoc.201501614

[B24] Eyckens D. J. Demir B. Walsh T. R. Welton T. Henderson L. C. (2016b). Determination of Kamlet–taft parameters for selected solvate ionic liquids. Phy. Chem. Chemical Phy. 18, 13153–13157. 10.1039/C6CP01216G 27122349

[B25] Eyckens D. J. Henderson L. C. (2017). Synthesis of α-aminophosphonates using solvate ionic liquids. RSC Adv. 7, 27900–27904. 10.1039/C7RA04407K

[B26] Eyckens D. J. Servinis L. Scheffler C. Wolfel E. Demir B. Walsh T. R. (2018). Synergistic interfacial effects of ionic liquids as sizing agents and surface modified carbon fibers. J. Mater. Chem. A. 6, 4504–4514. 10.1039/C7TA10516A

[B27] Ferrara C. V. Dall'Asta Berbenni V. Quartarone E. Mustarelli P. (2017). Physicochemical characterization of AlCl3–1-Ethyl-3-methylimidazolium chloride ionic liquid electrolytes for aluminum rechargeable batteries. J. Phy. Chem. C. 121, 26607–26614. 10.1021/acs.jpcc.7b07562

[B28] Fields E. K. (1952). The synthesis of esters of substituted amino phosphonic acids. J. Am. Chem. Soc. 74, 1528–1531. 10.1021/ja01126a054

[B29] Forman M. A. Dailey W. P. (1991). The lithium perchlorate-diethyl ether rate acceleration of the Diels-Alder reaction: Lewis acid catalysis by lithium ion. J. Am. Chem. Soc. 113, 2761–2762. 10.1021/ja00007a065

[B30] Forsyth C. M. MacFarlane D. R. Golding J. J. Huang J. Sun J. Forsyth M. (2002). Structural characterization of novel ionic materials incorporating the Bis(trifluoromethanesulfonyl)amide anion. Chem. Mater. 14, 2103–2108. 10.1021/cm0107777

[B31] Fujinami T. Buzoujima Y. (2003). Novel lithium salts exhibiting high lithium ion transference numbers in polymer electrolytes. J. Power Sources 119-121, 438–441. 10.1016/S0378-7753(03)00185-X

[B32] Grieco P. A. Moher E. D. (1993). Lithium catalyzed hetero Diels-Alder reactions cyclocondensation of N-protected α-amino aldehydes with 1-methoxy-3-tert-butyldimethylsilyloxybutadiene in the presence of lithium perchlorate. Tetrahedron Lett. 34, 5567–5570. 10.1016/S0040-4039(00)73883-4

[B33] Grieco P. A. Nunes J. J. Gaul M. D. (1990). Dramatic rate accelerations of Diels-Alder reactions in 5 M lithium perchlorate-diethyl ether: the cantharidin problem reexamined. J. Am. Chem. Soc. 112, 4595–4596. 10.1021/ja00167a096

[B34] Guo Y.-C. Li J. J. Ma L. Z. Yu R. H. Wang W. W. Zhu J. X. (2015). Synthesis and antitumor activity of α-aminophosphonate derivatives containing thieno[2,3-d]pyrimidines. Chin. Chem. Lett. 26, 755–758. 10.1016/j.cclet.2015.03.026

[B35] Gutmann V. (1976). Solvent effects on the reactivities of organometallic compounds. Coordinat. Chem. Rev. 18, 225–255. 10.1016/S0010-8545(00)82045-7

[B36] Hallett J. P. Welton T. (2011). Room-temperature ionic liquids: solvents for synthesis and catalysis. Chem. Rev. 111, 3508–3576. 10.1021/cr1003248 21469639

[B37] Hawker R. R. Haines R. S. Harper J. B. (2018). The effect of varying the anion of an ionic liquid on the solvent effects on a nucleophilic aromatic substitution reaction. Org. Biomol. Chem. 16, 3453–3463. 10.1039/C8OB00651B 29683173

[B38] Henderson W. A. (2006). Glyme–lithium salt phase behavior. J. Phys. Chem. B. 110, 13177–13183. 10.1021/jp061516t 16805630

[B39] Henderson W. A. Brooks N. R. Young V. G. (2003). Tetraglyme–Li+ cation solvate structures: models for amorphous concentrated liquid and polymer electrolytes (II). Chem. Mater. 15, 4685–4690. 10.1021/cm034352r

[B40] Heo Y. Cho D. H. Mishra M. K. Jang D. O. (2012). Efficient one-pot synthesis of α-aminophosphonates from aldehydes and ketones catalyzed by ytterbium(III) triflate. Tetrahedron Lett. 53, 3897–3899. 10.1016/j.tetlet.2012.05.068

[B41] Heydari A. (2002). Organic synthesis in an unconventional solvent, 5.0 M lithium perchlorate/diethyl ether. Tetrahedron 58, 6777–6793. 10.1016/S0040-4020(02)00745-7

[B42] Hirakimoto T. Nishiura M. Watanabe M. (2001). Effects of addition of a boric acid ester monomer to electrolyte solutions and gel electrolytes on their ionic transport properties. Electrochim. Acta 46, 1609–1614. 10.1016/S0013-4686(00)00760-X

[B43] Kabachnik M. I. M. Ya T. (1952). A new method for the synthesis of α-amino phosphoric acids. Doklady Akademii Nauk 83:689.

[B44] Kamlet M. J. Abboud J. L. Taft R. W. (1977). The solvatochromic comparison method. *6*. The .pi.^*^ scale of solvent polarities. J. Am. Chem. Soc. 99, 6027–6038.

[B45] Kamlet M. J. Taft R. W. (1976). The solvatochromic comparison method. I. The .beta.-scale of solvent hydrogen-bond acceptor (HBA) basicities. J. Am. Chem. Soc. 98, 377–383.

[B46] Kawazoe T. Hashimoto K. Kitazawa Y. Kokubo H. Watanabe M. (2017). A Polymer electrolyte containing solvate ionic liquid with increased mechanical strength formed by self-assembly of ABA-type ionomer triblock copolymer. Electrochim. Acta 235, 287–294. 10.1016/j.electacta.2017.03.125

[B47] Keaveney S. T. Haines R. S. Harper J. B. (2017). Investigating solvent effects of an ionic liquid on pericyclic reactions through kinetic analyses of simple rearrangements. ChemPlusChem. 82, 449–457. 10.1002/cplu.201600585 31962019

[B48] Kido R. Ueno K. Iwata K. Kitazawa Y. Imaizumi S. Mandai T. (2015). Li+ ion transport in polymer electrolytes based on a glyme-Li salt solvate ionic liquid. Electrochim. Acta 175, 5–12. 10.1016/j.electacta.2015.01.067

[B49] Krampa F. D. Aniweh Y. Awandare G. A. Kanyong P. (2017). A Disposable amperometric sensor based on high-performance PEDOT:PSS/ionic liquid nanocomposite thin film-modified screen-printed electrode for the analysis of catechol in natural water samples. Sensors 17:1716. 10.3390/s17081716 28933756 PMC5579879

[B50] Lee J.-M. Ruckes S. Prausnitz J. M. (2008). Solvent polarities and kamlet–taft parameters for ionic liquids containing a pyridinium cation. J. Phys. Chem. B. 112, 1473–1476. 10.1021/jp076895k 18201076

[B51] Lee J. H. Kim W. H. Danishefsky S. J. (2009). Syntheses of isomerically pure reference octalins and hydrindanes. Tetrahedron Lett. 50, 5482–5484. 10.1016/j.tetlet.2009.07.068 20711484 PMC2919757

[B52] Li H. Rutland M. W. Watanabe M. Atkin R. (2017). Boundary layer friction of solvate ionic liquids as a function of potential. Faraday Discuss. 199, 311–322. 10.1039/C6FD00236F 28422196

[B53] Li J. Wang Y. Sun Y. Ding C. Lin Y. Sun W. Luo C. (2017). A novel ionic liquid functionalized graphene oxide supported gold nanoparticle composite film for sensitive electrochemical detection of dopamine. RSC Adv. 7, 2315–2322. 10.1039/C6RA25627A

[B54] Li X.-C. S. Gong S. D.-Zeng Y. Y. You H. Sun Q. (2016). Highly efficient synthesis of α-aminophosphonates catalyzed by hafnium(IV) chloride. Tetrahedron Lett. 57, 1782–1785. 10.1016/j.tetlet.2016.03.033

[B55] Liu N. Luo F. Wu H. Liu Y. Zhang C. Chen J. (2008). One-step ionic-liquid-assisted electrochemical synthesis of ionic-liquid-functionalized graphene sheets directly from graphite. Adv Func Mater. 18, 1518–1525. 10.1002/adfm.200700797

[B56] Mandai T. Yoshida K. Ueno K. Dokko K. Watanabe M. (2014). Criteria for solvate ionic liquids. Phys. Chem. Chem. Phys. 16, 8761–8772. 10.1039/c4cp00461b 24676567

[B57] Mary Sukeshini A. Nishimoto A. Watanabe M. (1996). Transport and electrochemical characterization of plasticized poly(vinyl chloride) solid electrolytes. Solid State Ionics, 86–88, 385–393. 10.1016/0167-2738(96)00156-7

[B58] Mayer U. Gutmann V. Gerger W. (1975). The acceptor number — a quantitative empirical parameter for the electrophilic properties of solvents. Monatshefte für Chemie. 106, 1235–1257. 10.1007/BF00913599

[B59] Muldoon M. J. Gordon C. M. Dunkin I. R. (2001). Investigations of solvent-solute interactions in room temperature ionic liquids using solvatochromic dyes. *J. Chem*. Soc. Perkin Trans. 2, 433–435. 10.1039/b101449h

[B60] Murphy T. Callear S. K. Yepuri N. Shimizu K. Watanabe M. Canongia Lopes J. N. (2016). Bulk nanostructure of the prototypical ‘good' and ‘poor' solvate ionic liquids [Li(G4)][TFSI] and [Li(G4)][NO3]. Phys. Chem. Chem. Phy. 18, 17224–17236. 10.1039/c6cp00176a. 26845292

[B61] Musiał M. Malarz K. Mrozek-Wilczkiewicz A. Musiol R. Zorebski E. Dzida M. (2017). Pyrrolidinium-based ionic liquids as sustainable media in heat-transfer processes. ACS Sustain. Chem. Eng. 5, 11024–11033. 10.1021/acssuschemeng.7b02918

[B62] Nakazawa T. Ikoma A. Kido R. Ueno K. Dokko K. Watanabe M. (2016). Effects of compatibility of polymer binders with solvate ionic liquid electrolytes on discharge and charge reactions of lithium-sulfur batteries. J. Power Sources. 307, 746–752. 10.1016/j.jpowsour.2016.01.045

[B63] Nishimoto A. Watanabe M. Ikeda Y. Kohjiya S. (1998). High ionic conductivity of new polymer electrolytes based on high molecular weight polyether comb polymers. Electrochimica Acta 43, 1177–1184. 10.1016/S0013-4686(97)10017-2

[B64] Obregón-Zúñiga A. Milán M. Juaristi E. (2017). Improving the catalytic performance of (S)-proline as organocatalyst in asymmetric aldol reactions in the presence of solvate ionic liquids, involvement of a supramolecular aggregate. Org. Lett. 19, 1108–1111. 10.1021/acs.orglett.7b00129 28199118

[B65] Padró J. M. Reta M. (2016). Solvatochromic parameters of imidazolium-, hydroxyammonium-, pyridinium- and phosphonium-based room temperature ionic liquids. J. Mol. Liquids 213, 107–114. 10.1016/j.molliq.2015.10.055

[B66] Pappenfus T. M. Henderson W. A. Owens B. B. Mann K. R. Smyrl W. H. (2004). Complexes of lithium imide salts with tetraglyme and their polyelectrolyte composite materials. J. Electrochem. Soc. 151, A209–A215. 10.1149/1.1635384

[B67] Peng J. Carbone L. Gobet M. Hassoun J. Devany M. Greenbaum S. (2016). Natural Abundance oxygen-17 NMR investigation of lithium ion solvation in glyme-based electrolytes. Electrochimica Acta. 213, 606–612. 10.1016/j.electacta.2016.07.144

[B68] Pettersen D. Marcolini M. Bernardi L. Fini F. Herrera R. P. Sgarzani V.. (2006). Direct access to enantiomerically enriched α-amino phosphonic acid derivatives by organocatalytic asymmetric hydrophosphonylation of imines. J. Org. Chem. 71, 6269–6272. 10.1021/jo060708h 16872218

[B69] Qureshi Z. Deshmukh K. Bhanage B. (2014). Applications of ionic liquids in organic synthesis and catalysis. *Clean Technol. Environ*. Policy. 16, 1487–1513. 10.1007/s10098-013-0660-0

[B70] Reichardt C. (1994). Solvatochromic dyes as solvent polarity indicators. Chem. Rev. 94, 2319–2358. 10.1021/cr00032a005

[B71] Renninger N. Mcphee D. J. (2008). Fuel Compositions Comprising Farnesane and Farnesane Derivatives and Method of Making and Using Same. US. WO/2008/045555.

[B72] Reta M. Cattana R. Silber J. J. (2001). Kamlet–Taft's solvatochromic parameters for nonaqueous binary mixtures between n-hexane and 2-propanol, tetrahydrofurane, and ethyl acetate. J. Sol. Chem. 30, 237–252. 10.1023/A:1005275432313

[B73] Saito S. Watanabe H. Hayashi Y. Matsugami M. Tsuzuki S. Seki S. (2016). Li+ Local structure in Li–tetraglyme solvate ionic liquid revealed by neutron total scattering experiments with the 6/7Li isotopic substitution technique. J. Phy. Chem. Lett. 7, 2832–2837. 10.1021/acs.jpclett.6b01266 27388117

[B74] Seki S. Takei K. Miyashiro H. Watanabe M. (2011). Physicochemical and electrochemical properties of glyme-LiN(SO2F)2 complex for safe lithium-ion secondary battery electrolyte. J. Electrochem. Soc. 158, A769–A774. 10.1149/1.3582822

[B75] Shan C. Yang H. Han D. Zhang Q. Ivaska A. Niu L. (2010). Electrochemical determination of NADH and ethanol based on ionic liquid-functionalized graphene. Biosens. Bioelectron. 25, 1504–1508. 10.1016/j.bios.2009.11.009 20007014

[B76] Shobukawa H. Tokuda H.S.-Tabata I. Watanabe M. (2004). Preparation and transport properties of novel lithium ionic liquids. Electrochim. Acta. 50, 305–309. 10.1016/j.electacta.2004.01.096

[B77] Springer G. Elam C. Edwards A. Bowe C. Boyles D. Bartmess J. (1999). Chemical and spectroscopic studies related to the lewis acidity of lithium perchlorate in diethyl ether. J. Org. Chem. 64, 2202–2210. 10.1021/jo981042x

[B78] Tabata S.-i. Hirakimoto T. Nishiura M. Watanabe M. (2003). Synthesis of a Lewis-acidic boric acid ester monomer and effect of its addition to electrolyte solutions and polymer gel electrolytes on their ion transport properties. Electrochim. Acta 48, 2105–2112. 10.1016/S0013-4686(03)00192-0

[B79] Taft R. W. Kamlet M. J. (1976). The solvatochromic comparison method. 2. The .alpha.-scale of solvent hydrogen-bond donor (HBD) acidities. J. Am. Chem. Soc. 98, 2886–2894.

[B80] Tamura T. Yoshida K. Hachida T. Tsuchiya M. Nakamura M. Kazue Y. (2010). Physicochemical properties of glyme-Li salt complexes as a new family of room-temperature ionic liquids. Chem. Lett. 39, 753–755. 10.1246/cl.2010.753

[B81] Ueno K. Tatara R. Tsuzuki S. Saito S. Doi H. Yoshida K.. (2015). Li+ solvation in glyme-Li salt solvate ionic liquids. Phy. Chem. Chem. Phys. 17, 8248–8257. 10.1039/C4CP05943C 25733406

[B82] Ueno K. Yoshida K. Tsuchiya M. Tachikawa N. Dokko K. Watanabe M. (2012). Glyme–lithium salt equimolar molten mixtures, concentrated solutions or solvate ionic liquids? J. Phy. Chem. B. 116, 11323–11331. 10.1021/jp307378j 22897246

[B83] Ueno K. J. Park W. Yamazaki A. Mandai T. Tachikawa N. Dokko K. (2013). Anionic effects on solvate ionic liquid electrolytes in rechargeable lithium–sulfur batteries. J. Phy. Chem. C. 117, 20509–20516. 10.1021/jp407158y

[B84] Wang Y. Li C. Wu T. Ye X. (2018). Polymerized ionic liquid functionalized graphene oxide nanosheets as a sensitive platform for bisphenol A sensing. Carbon 129, 21–28. 10.1016/j.carbon.2017.11.090

[B85] Wasserscheid P. Keim W. (2000). Ionic liquids—new solutions for transition metal catalysis. Angew. Chem. Int. Ed. 39, 3772–3789. 10.1002/1521-3773(20001103)39:21<3772::AID-ANIE3772>3.0.CO;2-5 11091453

[B86] Watanabe M. Mizumura T. (1996). Conductivity study on ionic liquid/polymer complexes. Solid State Ionics, 86-88, 353–356. 10.1016/0167-2738(96)00136-1

[B87] Watanabe M. Nishimoto A. (1995). Effects of network structures and incorporated salt species on electrochemical properties of polyether-based polymer electrolytes. Solid State Ionics, 79, 306–312. 10.1016/0167-2738(95)00079-L

[B88] Welton T. (1999). Room-temperature ionic liquids. solvents for synthesis and catalysis. Chem. Rev. 99, 2071–2084. 10.1021/cr980032t 11849019

[B89] Zad Z. R. Davarani S. S. H. Taheri A. Bide Y. (2018). A yolk shell Fe3O4 @PA-Ni@Pd/Chitosan nanocomposite -modified carbon ionic liquid electrode as a new sensor for the sensitive determination of fluconazole in pharmaceutical preparations and biological fluids. J. Mol. Liq. 253, 233–240. 10.1016/j.molliq.2018.01.019

[B90] Zhang Q. Wu S. Zhang L. Lu J. Verproot F. Liu Y.. (2011). Fabrication of polymeric ionic liquid/graphene nanocomposite for glucose oxidase immobilization and direct electrochemistry. Biosen. Bioelectron. 26, 2632–2637. 10.1016/j.bios.2010.11.024 21159504

